# Strain engineering of 2D semiconductors and graphene: from strain fields to band-structure tuning and photonic applications

**DOI:** 10.1038/s41377-020-00421-5

**Published:** 2020-11-23

**Authors:** Zhiwei Peng, Xiaolin Chen, Yulong Fan, David J. Srolovitz, Dangyuan Lei

**Affiliations:** 1 Department of Materials Science and Engineering, City University of Hong Kong, 83 Tat Chee Avenue, Kowloon, Hong Kong, 999077 China; 2 Department of Applied Biology and Chemical Technology, The Hong Kong Polytechnic University, Hung Hom, Hong Kong, 999077 China

**Keywords:** Optical properties and devices, Photonic devices

## Abstract

Two-dimensional (2D) transition metal dichalcogenides (TMDCs) and graphene compose a new family of crystalline materials with atomic thicknesses and exotic mechanical, electronic, and optical properties. Due to their inherent exceptional mechanical flexibility and strength, these 2D materials provide an ideal platform for strain engineering, enabling versatile modulation and significant enhancement of their optical properties. For instance, recent theoretical and experimental investigations have demonstrated flexible control over their electronic states via application of external strains, such as uniaxial strain and biaxial strain. Meanwhile, many nondestructive optical measurement methods, typically including absorption, reflectance, photoluminescence, and Raman spectroscopies, can be readily exploited to quantitatively determine strain-engineered optical properties. This review begins with an introduction to the macroscopic theory of crystal elasticity and microscopic effective low-energy Hamiltonians coupled with strain fields, and then summarizes recent advances in strain-induced optical responses of 2D TMDCs and graphene, followed by the strain engineering techniques. It concludes with exciting applications associated with strained 2D materials, discussions on existing open questions, and an outlook on this intriguing emerging field.

## Optics: 2D materials feel the strain

A review of recent advances in strain-induced new optical responses of two-dimensional (2D) materials concludes with various applications associated with strain-engineered 2D materials. The review, conducted by Dangyuan Lei and colleagues at The City University of Hong Kong, in collaboration with a researcher from The Hong Kong Polytechnic University, first provides a brief introduction to the macroscopic theory of crystal elasticity and how external strain affects the physical and optical properties of 2D materials. It then summarizes how recent advances in the application of external strains in 2D materials, including TMDCs and graphene, can be used to modify their unique physical and optical properties, followed by a summary of strain engineering techniques. The review concludes by highlighting some of the peculiar applications associated with strained 2D materials, such as high-sensitivity optical resonators and flexible electronic devices.

## Introduction

Graphene, the earliest discovered two-dimensional (2D) material (in 2004)^1^, which flakes from bulk graphite via mechanical exfoliation, opened the door to the investigation of a large variety of 2D materials within the scientific and technological community^2–4^. These layered materials have strong covalent in-plane bonding yet weak interlayer van der Waals interactions. The 2D nature of these monolayer materials endows them with unique electronic structures, and extraordinary physical and chemical properties. For example, charge carriers in graphene exhibit massless Dirac fermion behavior with relativistic particles traveling at near the “speed of light” (10^6^ m/s) near the gapless K and K′ points in the Brillouin zone (BZ)^5^. However, the lack of an intrinsic optical bandgap makes pristine graphene far less appealing for light-emitting applications since zero-bandgap materials do not emit light under continuous-wave excitation (except under ultrafast laser pulses, ~30 fs)^6^. Hence, one of the most powerful optical characterization techniques for graphene is Raman spectroscopy, which involves inelastic scattering of photons that allows monitoring of doping, defect, disorder, and edge site effects^7^.

In addition to the tremendous theoretical and experimental advances in graphene research over the past decade, many additional 2D materials with unprecedented properties have been discovered and studied^8^. Among them, the 2D group-VI transition metal dichalcogenides (TMDCs) represent a class of promising materials that are candidates for overcoming some of the disadvantages of graphene for future applications, such as ultrathin, flexible photonic, and optoelectronic devices that require optically transparent semiconductors^9^. TMDC monolayers of the MX_2_ form have a hexagonal layer of metal atoms (M) sandwiched between atomic layers of chalcogen atoms (X). The X-M-X layers can be stacked to form van der Waals-bonded, multilayered TMDCs. While consideration of MX_2_ TMDCs with X = {S, Se, Te} and M = {Mo, W, Re, V} implies 12 different types of TMDCs^10^, the Janus structures of the X_1_-M-X_2_ type (i.e., different chalcogens in the nonmetal layers) yield 24 more TMDCs^11^, and alloying within the M- and X-layers produces an infinite set of possible TMDCs. When a TMDC monolayer is extracted from a bulk TMDC crystal, the bandgap may change from indirect-to-direct via the inversion symmetry breaking, resulting in an increase in the optical bandgap because of the significantly enhanced Coulomb interaction arising from the reduction in the dielectric screening effect (e.g., 1.9 eV for monolayer MoS_2_ compared to 1.29 eV for bulk MoS_2_^12,13^). Because of these distinctive electronic properties (mostly absent in graphene), TMDC monolayers exhibit prominent photoluminescence (PL) with composition- and layer number-dependent emission energies, thereby enriching their optical properties and opening up new optoelectronic applications. Theoretical calculations and subsequent experimental demonstrations have also shown direct bandgaps in other TMDCs, such as MoSe_2_, WS_2_, and WSe_2_ (ref. ^14^). These direct bandgaps in TMDC monolayers, together with their spin-valley-coupling-induced valleytronic effects^15–17^, have triggered a plethora of potential optoelectronic applications, such as field-effect transistors, logic circuits, amplifiers, photodetectors, and excitonic light-emitting devices^9,18–21^. The weak interactions between 2D monolayers provide an opportunity for easy heterostructure design via vertical stacking of different 2D materials (graphene, hexagonal boron nitride, TMDCs, etc.)^22–26^. 2D-material-based heterojunctions/stacks also provide an effective means of tuning optical properties and an atomic-precision, semiconducting building block device design/assembly approach, which endows these materials with the potential for integration with advanced optical platforms, such as microcavities^27^ and metasurfaces^17^, to realize versatile functionalities.

Recent theoretical and experimental work has demonstrated that strain can also play an important role in manipulating the electronic and optical properties of TMDCs and graphene^28–32^. The remarkable strength of TMDCs^33^, compared with their conventional semiconducting counterparts^34–36^, allows the application of large strains for band structure manipulation. In 1921, Griffith argued that a perfect (defect-free), elastic crystal has a theoretical fracture strength approximately one-ninth its Young’s modulus^37^. Motivated by this insight, researchers sought this theoretical upper limit of the fracture strength in the fertile ground of defect-free, atomically thick, 2D materials. For example, density-functional theory (DFT) calculations and atomic force microscope (AFM) tip-based experiments showed that the fracture stress of a freely suspended MoS_2_ monolayer is approximately one-eighth of its Young’s modulus, which approaches the theoretical limit suggested by Griffith theory^34,35,37,38^. For example, MoS_2_ monolayers show a maximum strain at fracture of >10%, whereas bulk silicon usually breaks at a strain of ≤1.5% (ref. ^39^). While application of macroscopically homogeneous strains on a large scale are achievable, inhomogeneous strains provide a means of locally creating spatially varying band structures within 2D TMDCs on the nanoscale. For example, wrinkles in MoS_2_ produce such locally varying strains^40,41^. Similar to 2D TMDCs, graphene can also sustain very large elastic strains (up to 25%), comparable to Griffith’s theoretical limit (note that the Young’s modulus of graphene is much greater than that of monolayer TMDCs). As a consequence, graphene is also expected to be an excellent platform for the study of strain engineering in 2D materials^42^.

The remarkable strain limit of 2D TMDCs and graphene provides a straightforward, effective means of continuously tuning electronic and optical properties; this has led to the bourgeoning research on optoelectronic devices based on strain-induced modification of optical and electronic properties^39^. Many intriguing theoretical studies and experimental measurements of strained 2D materials have demonstrated how strains modify electronic and optical properties. For example, a direct-to-indirect bandgap transition and a semiconductor-to-metal phase transition are expected to occur in monolayer MoS_2_ under ~2% uniaxial tensile strain and 10–15% biaxial tensile strain, respectively^43–45^. Theoretically, strain-induced changes in the optical bandgap of 2D TMDCs (larger than 1 eV)^43^ enable nondestructive spectroscopic investigations of the effect of different components of strain on their mechanical, electronic, optical, and chemical properties. Similarly, the electronic and optical properties of strained graphene also strongly depend on the strain distribution. In fact, a large strain can drastically modify the electronic band structure of graphene, and even give rise to the appearance of an optical bandgap^46^ and a significant change in the optical conductivity^47^. Therefore, strain engineering of both 2D TMDCs and graphene can play a significant role in understanding the electronic and optical properties of 2D materials, and their device applications.

Since many review articles have summarized the fundamental properties^48,49^, synthesis approaches^3,48,49^, and optoelectronics applications^48–51^ of unstrained TMDCs and graphene, in this review, we mainly concentrate on the effects of strain on intrinsic optical properties, strain-induced optical effects, and their applications. We first present an overview of the linear theory of elasticity and effective low-energy Hamiltonians to facilitate the understanding of the peculiar properties induced by strains in TMDCs and graphene. Next, we introduce both optical and electronic properties of pristine TMDCs and graphene, as well as strain-induced optical effects, such as the tuneable PL of TMDCs and Raman modes of graphene under different types of strains. Then, we describe some current strain engineering techniques, and consider their applications and achievable strain levels. Finally, we discuss a series of important photonic applications of strain-induced optical effects in 2D TMDCs and graphene, and provide an outlook for future work in this fast-growing area.

## Theory of elasticity and an effective low-energy Hamiltonian

Quantum theory provides a powerful theoretical tool to deeply understand various physical phenomena in emerging semiconducting crystals. The resulting electronic band structure and wave functions can be employed to theoretically calculate observable physical quantities, e.g., the spontaneous emission rate with the aid of Fermi’s golden rule^52^, which in turn accounts for the light absorption and emission induced by optical interband transitions of electrons. Several effective approaches have been introduced to quantitatively understand the physical properties of strained 2D materials, including DFT and symmetry-allowed low-energy effective fields based on group theory ideas^53,54^. In the absence of an external strain, graphene and most TMDC monolayers share the same hexagonal Bravais lattice characterized by primitive unit cells plus a set of basis atoms. Mathematically, the two primitive lattice vectors, designated ***a***_1_ and ***a***_2_ can be expressed by a linear combination of bond vectors between adjacent atoms, ***δ***_*n*_, such as three and six nearest-neighbor vectors for graphene and TMDCs, respectively^55^. However, when the 2D materials are strained, the Bravais lattices are deformed. At the same time, the modified bond vectors can be exploited to parameterize the valence force models to describe the elastic energy of strained 2D materials^56^. On the other hand, from a macroscopic point of view, the 2D materials deformed under external applied strains can be described by continuum elasticity theory, provided that the length scale of the deformation is large compared with the lattice constant^54^. The elastic energy stored in 2D materials can be divided into stretching and bending energies. In such a configuration, a generic atom in the deformed crystalline structure of a 2D material whose original position is ***r*** = (*x, y*) experiences a displacement ${\boldsymbol{u}}\left( {\boldsymbol{r}} \right) + \hat zh\left( {\boldsymbol{r}} \right),$ where ${\boldsymbol{u}}\left( {\boldsymbol{r}} \right) = \left( {u_x\left( {\boldsymbol{r}} \right),u_y\left( {\boldsymbol{r}} \right)} \right)$ is a 2D vector field describing the in-plane deformation, and *h*(***r***) is a scalar field accounting for out-of-plane deformations. The strain is a second-rank tensor field

1 $$\varepsilon _{ij} = \frac{1}{2}\left( {\partial _iu_j + \partial _ju_i + \partial _ih\partial _jh} \right)$$

where *i, j* ∈ {*x, y*}. The elastic energy density *F*_el_ (a contribution to the potential energy of the Hamiltonian) of anisotropic membrane can be written as a sum of the stretching energy density *F*_st_ (energy cost due to in-plane relative distance changes) and the bending energy density *F*_b_ (resulting from deviations from the flat configuration), as described in the following equations:

2 $$F_{{\mathrm{el}}} = F_{{\mathrm{st}}} + F_{\mathrm{b}},{\mathrm{with}}\,F_{{\mathrm{st}}} = \frac{1}{2}\left( {\lambda \varepsilon _{ii}^2 + 2\mu \varepsilon _{ij}\varepsilon _{ij}} \right)\,{\mathrm{and}}\,F_{\mathrm{b}} = \frac{1}{2}\kappa \left( {\nabla ^2h} \right)^2$$

where *λ* and *μ* are the (in-plane) Lamé constants of the material and *κ* is the bending rigidity. The stretching energy density can be explicitly written as^55^

3 $$F_{{\mathrm{st}}} = \frac{{Y_{2d}}}{{2\left( {1 - \nu ^2} \right)}}\left[ {\varepsilon _{xx}^2 + \varepsilon _{yy}^2 + 2\nu \varepsilon _{xx}\varepsilon _{yy} + \left( {1 - \nu } \right)\varepsilon _{xy}^2} \right]$$

where *Y*_2*d*_ and *v* are the 2D Young’s modulus and Poisson ratio, respectively, which can be expressed in terms of the Lamé constants as

4 $$Y_{2d} = \left( {\lambda + 2\mu } \right)\left( {1 - \nu ^2} \right)\,{\mathrm{and}}\,\nu = \frac{\lambda }{{\lambda + 2\mu }}$$

Here, it is worth noting that the Lamé constants and bending rigidity can be related to the microscopic parameters of 2D materials through the valence force field^55^. Thus, Eqs. (2)–(4) allow the development of a bridge between macroscopic strains and microscopic parameters of the materials. We apply these equations to determine the electronic band structure by constructing microscopic Hamiltonians in the tight-binding (TB) and ***k ⋅ p*** models through the strain-displacement relations obtained by minimizing the elastic energy *F*_el_ for a given strain (i.e., mechanical equilibrium). More specifically, for the non-Bravais lattices of strained materials, the strain-dependent first-neighbor vector ${{\updelta}}^{\prime}_n = \left( {{\boldsymbol{I}} + \varepsilon } \right) \cdot {{\updelta }}_n$, where *ε* is the strain tensor and relates to the displacement via ***u***(***r***) = *ε* · ***r***, breaks down within the Cauchy–Born approximation^53^ and should be corrected as ${{\updelta}}^{\prime}_n = \left( {{\boldsymbol{I}} + \varepsilon } \right) \cdot {{\updelta }}_n + \Delta$, where the additional displacement Δ accounts for the additional degrees of freedom introduced by multiple basis atoms in the primitive unit cell. The vector Δ is determined by minimizing the deformation energy of the material. In this way, the TB Hamiltonian incorporating first-neighbor vectors can be used to deduce strain-related effects in a graphene lattice^53^

5 $$H = - \mathop {\sum}\limits_{{\boldsymbol{r}}^\prime} \mathop{\sum}\limits_{n = 1}^3t_{{\boldsymbol{r}}^\prime,{{\updelta}}_{\boldsymbol{n}}^\prime }a_{{\boldsymbol{r}}^\prime}^\dagger b_{{\boldsymbol{r}}^\prime + {{\updelta }}_{\boldsymbol{n}}^\prime} + {\mathrm{H}}.{\mathrm{c}}.$$

where H.c. is the Hermitian conjugate, $t_{{\boldsymbol{r}}^\prime ,{\boldsymbol{\delta }}_{\boldsymbol{n}}^\prime }$ indicates the hopping integral in the deformed lattice, and $a_{{\boldsymbol{r}}^\prime }^\dagger$ and $b_{{\boldsymbol{r}}^\prime + {\boldsymbol{\delta }}_{\boldsymbol{n}}^\prime }$ are electron creation and annihilation operators on the A sublattice (at position ***r***′) and the B sublattice (at position ${\boldsymbol{r}}^\prime + {\boldsymbol{\delta }}_{\boldsymbol{n}}^\prime$), respectively.

In practice, the TB model discussed above is widely applied to explore the strain-induced energy landscape of graphene^57,58^ (Fig. 5a), while the DFT method is used to determine the effect of strain on the band structure of 2D TMDCs (Fig. 2a). However, the application of DFT methods is computationally prohibitive when the elastic strains are inhomogeneous. In such situations, the TB model provides a useful approach to evaluate the effects of strain on the band structure of 2D materials under inhomogeneous strains (Fig. 3a).

Recently, other effective Hamiltonians with coupling terms related to external fields (e.g., strain and electromagnetic fields), have been proposed to gain a better understanding of the physics behind novel effects in 2D materials (e.g., those associated with strains and valley Hall effects)^59^. One way to construct such effective Hamiltonians that account for strain and electron momentum is through the crystal symmetry group of 2D materials, and their corresponding irreducible representations. An alternative way of deriving effective Hamiltonians is based on direct expansion of the TB Hamiltonians. For example, in the presence of nonuniform lattice deformations, the low-energy effective Hamiltonian of graphene is given by^60^

6 $$H = H_0 + \mathop {\sum}\limits_{m = 1}^6 {a_mH_m} + \mathop {\sum}\limits_{m = 1}^6 {\tilde a_m\tilde H_m}$$

where $H_0 = \nu _F\left( { \pm \sigma _xk_x + \sigma _yk_y} \right)$ is the unstrained standard Dirac contribution (*v*_*F*_ and *σ*_*i*_, *i* ∈ {*x*, *y*}, are the Fermi velocity and Pauli matrices). The strained terms *a*_*m*_*H*_*m*_ and $\tilde a_m\tilde H_m$ relate to different effects induced by nonuniform strains^54^, such as the Dirac cone shift and tilt in momentum space and the gap-opening mechanism. As another example, the TB Hamiltonian of a uniformly strained TMDC (ignoring spin–orbit interactions) can be approximated by a reduced two-band model near the K points consisting of the highest valence band and the lowest conduction band^59^. The total Hamiltonian is *H* = *H*_0_ + *H*_1_, where *H*_0_ is the effective Hamiltonian of the unstrained TMDC and the contribution of strains can be expressed by

7 $$H_1 = f_3\mathop {\sum}\limits_i {\varepsilon _{ii} + f_4} \mathop {\sum}\limits_i {\varepsilon _{ii}\sigma _z + f_5} \left[ {\left( {\varepsilon _{xx} - \varepsilon _{yy}} \right)\sigma _x - 2\varepsilon _{xy}\sigma _y} \right]$$

where *f*_3_, *f*_4_, and *f*_5_ represent the modification of the mid-gap position and pseudo-gauge field terms. Moreover, the spin–orbit couplings (1 ± *σ*_*z*_)*s*_*z*_ and out-of-plane deformations can be accounted for by corrections to these Hamiltonians.

The macroscopic and microscopic strain models, introduced above, can assist in understanding and predicting a wide range of optical and electronic properties of strained 2D materials, such as their PL and Raman spectra, as a result of interactions between photons, electrons, and phonons as a function of the (tensor) strain fields.

## Exotic optical properties of unstrained 2D TMDCs

**Fig. 1:**
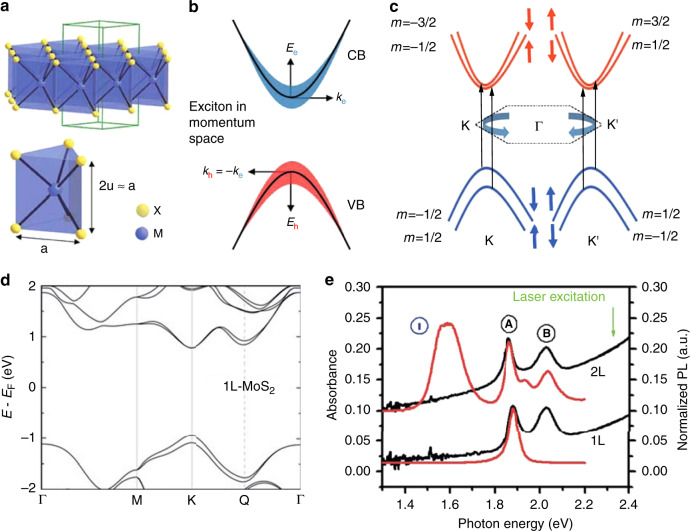
Crystal and electronic band structures, and PL and absorption spectra of unstrained 2D TMDCs. **a** Schematic of the crystal structure of monolayer MX_2_ (ref. ^14^). **b** Schematic of an exciton in reciprocal space formed via the Coulomb attraction between an electron in the conduction band (CB) and a hole in the valence band (VB)^62^. **c** Electronic bands around the K and K′ points of MX_2_ showing spin–orbit coupling-induced energy splitting. **d** Band structure of monolayer (1L) MoS_2_ obtained from DFT calculations considering spin–orbit coupling^14^. **e** Absorption spectra (black lines, normalized by the number of layers) and corresponding PL spectra (red lines, normalized by the intensity of peak A) of monolayer (1L) and bilayer (2L) MoS_2_ (ref. ^12^). Reprinted **a** and **d** from ref. ^14^ with permission from Wiley. Reprinted **b** with permission from ref. ^62^. Copyright (2018) by the American Physical Society. Reprinted **e** with permission from ref. ^12^. Copyright (2010) by the American Physical Society

TMDCs exhibit brilliant PL and absorption spectra because of their excellent luminescent properties (stemming from their large intrinsic bandgaps), which can be used to investigate and characterize their exotic optical properties. The unit cell of the crystal structure of monolayer TMDCs consists of transition metal atoms sandwiched between two layers of chalcogen atoms—typically forming a trigonal prismatic structure, as depicted in Fig. 1a. In general, the optical properties of TMDC monolayers are predominantly determined by the direct bandgap at the K and K′ points in the BZ, where the excitonic effect resulting from the strong Coulomb interaction between electron–hole pairs exists and plays a significant role in the optical spectrum resonance response (the sharp linewidth is determined by the lifetime of radiative excitons)^61,62^. We begin this section by briefly introducing some fundamental aspects of excitons in TMDCs.

An exciton, a hydrogen-like quasiparticle, is a bound state of an electron and a hole that are attracted to each other by the electrostatic Coulomb force, as depicted in Fig. 1b (refs. ^63–65^). This creates an optical bandgap significantly smaller than the electronic bandgap, e.g., 1.93 and 2.15 eV for the optical bandgap and electronic bandgap of 2D MoS_2_ (ref. ^66^). The discovery of A and B excitons in MoS_2_ in 2010 motivated researchers to focus on the fine structure of TMDC monolayers associated with electron spin^12^. The electronic spin–orbit interaction, originating from the *d* orbitals of heavy metal atoms, leads to splitting of both the conduction and valence bands. As a consequence, two direct optical transitions are allowed when the electron and hole states have identical spin, while the other two are forbidden due to the misalignment of their spin momenta at the K (K′) valley. The two optically allowed transitions are “bright excitons”, as shown in Fig. 1c (black arrows), and the other two are “dark excitons”. The electronic band landscape depicted in Fig. 1d unambiguously demonstrates the existence of A and B excitons associated with the spin splitting of the valence band.

The two degenerate K and K′ valleys in TMDC monolayers exhibit opposite spin momenta at the band edges. This and the spin splitting of the conduction and valence bands (originating from inversion symmetry breaking) give rise to the unique valley-dependent optical locking effect in these materials. Thus, the valley-dependent selection rules allow the binary information stored in each valley to be addressed by circularly polarized light, enabling potential applications in communications that exploit the valley pseudospin degree of freedom^17^. Evidence of A and B excitons in spectroscopy experiments has been seen in the absorption spectra of single and bilayer MoS_2_ (black lines in Fig. 1e).

Another tool broadly used to explore the optical properties of 2D materials is PL spectroscopy, as exemplified by the red curves in Fig. 1e for monolayer and bilayer MoS_2_, where A, B and, I represent the PL peaks at the A and B exciton and indirect bandgap point energies (the PL intensity of the B exciton in monolayer MoS_2_ is weak and can only be seen on a larger scale). Owing to the characteristic electronic properties, the absorption spectrum and PL spectrum are broadly exploited to quantitatively investigate the excitonic landscape, and distinguish different types of 2D TMDC^67,68^.

Raman spectroscopy is also exploited to identify the structure of 2D TMDCs through investigations of the interlayer van der Waals interaction, the electron–phonon interaction, and the long-range Columbic interaction between atoms^69–71^. For example, the phonon spectra of TMDCs are highly dependent on the applied strain, which can be seen by examining of the Raman peak shifts of the corresponding A_1g_ (out-of-plane) and E^1^_2g_ (in-plane) modes^72^. Optical spectroscopic characterization methods will be discussed in the following sections.

## Physics and new optical properties of strained 2D TMDCs

### Electronic band structures of strained 2D TMDCs

**Fig. 2:**
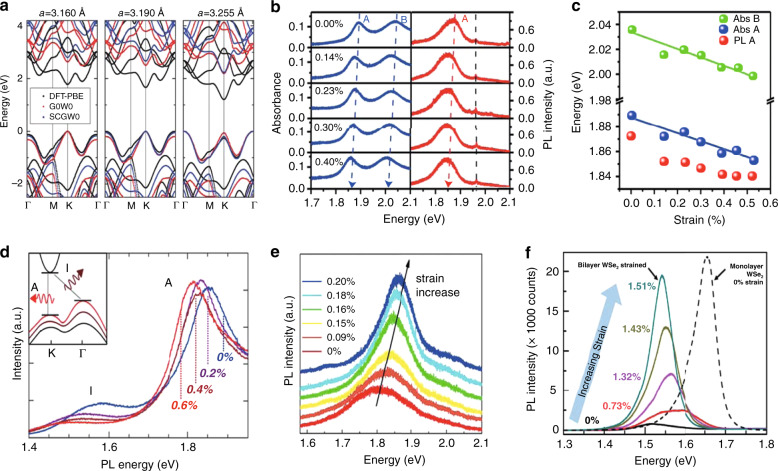
Band structures and PL and absorption spectra of strained TMDCs under different strains. **a** Band structures of monolayer MoS_2_ at lattice constant of 3.160, 3.190, and 3.225 Å corresponding to 0%, 1%, and 3% tensile strains calculated by DFT, G0W0, and SCGW0, respectively^32^. **b**, **c** Evolution of PL and absorption spectra (**b**) and peak positions (**c**) of monolayer MoS_2_ under various uniaxial tensile strains. Note that the dashed black line overlaid on the PL spectra in **b** indicates the PL peak of PMMA rather than that of the B exciton. Lines in **c** are linear fits to the data points extracted from **b**^29^. **d** PL spectra of bilayer MoS_2_ under uniaxial tensile strain increasing from 0 to 0.6%. The insert shows the indirect bandgap of bilayer MoS_2_ (ref. ^28^). **e** PL spectra of trilayer MoS_2_ under various biaxial compressive strains^76^. **f** PL spectra of bilayer WSe_2_ (solid lines) under various uniaxial tensile strains compared with that of unstrained monolayer WSe_2_ (dashed line)^78^. Reprinted **a** with permission from ref. ^32^. Copyright (2013) by the American Physical Society. Adapted **b**, **c** with permission from ref. ^29^. Copyright (2013) American Chemical Society. Adapted **d** with permission from ref. ^28^. Copyright (2013) American Chemical Society. Adapted **e** with permission from ref. ^76^. Copyright (2013) American Chemical Society. Reprinted **f** with permission from ref. ^78^. Copyright (2014) American Chemical Society

The intriguing optical properties of 2D materials are related to their complex electronic band structures^73^. As noted in the previous section, the dependence of the Hamiltonians of 2D materials on external strains can be used to identify strain-dependent effects in the electronic band structure. For example, both DFT and GW calculations predict a decrease in the bandgap in monolayer MoS_2_ with increasing tensile strain^32^, and suggest that a semiconductor-to-metal phase transition can occur under very large strains^43^. As illustrated in Fig. 2a, the impact of mechanical deformation on the electronic band structure is especially noticeable at the K point in the BZ, where the intrinsic direct bandgap decreases with increasing strain. Meanwhile, the top of the valence band shifts slightly upwards at the Γ point, stemming from the decrease in the orbital overlap between the metal and sulfur atoms associated with strain-induced changes in the bond distance. At sufficiently large strain, the valence band maximum moves from the K to Γ point, leading to a direct-to-indirect bandgap transition^32^. In addition to these uniform strain effects, wrinkling in MoS_2_ creates a nonuniform strain distribution that can reduce the direct bandgap (consistent with TB calculations)^41^.

### Strain tuning of the photoluminescence, absorption, and bandgap transition

As noted above, low-symmetry 2D TMDCs provide an excellent platform to continuously tune the electronic band structures and optical properties via external strains, as seen in many spectroscopy experiments^28,74,75^. For example, the peak positions in both the PL and absorption spectra of 2D TMDCs show a linear redshift for both the A and B excitons, when homogeneous tensile strain is applied (Fig. 2b). The experimental results shown in Fig. 2c reveal that the redshift rates in the absorption spectrum of monolayer MoS_2_ are −64 ± 5 meV/% (/% means per percent of tensile strain) for the A exciton and −68 ± 5 meV/% for the B exciton^29^. In addition, redshift rates of −54 ± 2 and −50 ± 3 meV/% are obtained for the A and B excitons in monolayer WSe_2_, respectively^74^. In addition to shifts in absorption peaks, PL spectral peaks also exhibit an approximately linear dependence on tensile strain^75^; for example, Fig. 2d shows redshift rates of −45 ± 7 meV/% for the A exciton in MoS_2_ monolayers, and −53 ± 10 meV/% for the A exciton and 129 ± 20 meV/% for the indirect bandgap (marked as “I” in the insert) in MoS_2_ bilayers^28^. Figure 2e shows a linear blueshift of the peak in trilayer MoS_2_ on a piezoelectric substrate with the application of a compressive biaxial strain^76^.

In addition to the redshift, when the strain is sufficiently large, a direct-to-indirect bandgap transition or an indirect-to-direct bandgap transition can be induced in TMDCs. For example, an indirect peak emerges when the strain is larger than 2.5% in strained WS_2_ monolayers^77^, while an indirect-to-direct transition occurs in strained WSe_2_ bilayers with an obvious enhancement of the PL intensity at 0.73% uniaxial tensile strain (Fig. 2f)^78^. Motivated by the aforementioned controllable optical properties, the variation in the measurable strain-related parameters with temperature^79^, input laser intensity^79^, and voltage^76^ can also be used to quantify the strains on TMDCs; this further broadens the potential optoelectronic, as well as strain sensor applications.

### Exciton funnel effect and spontaneous emission enhancement

**Fig. 3:**
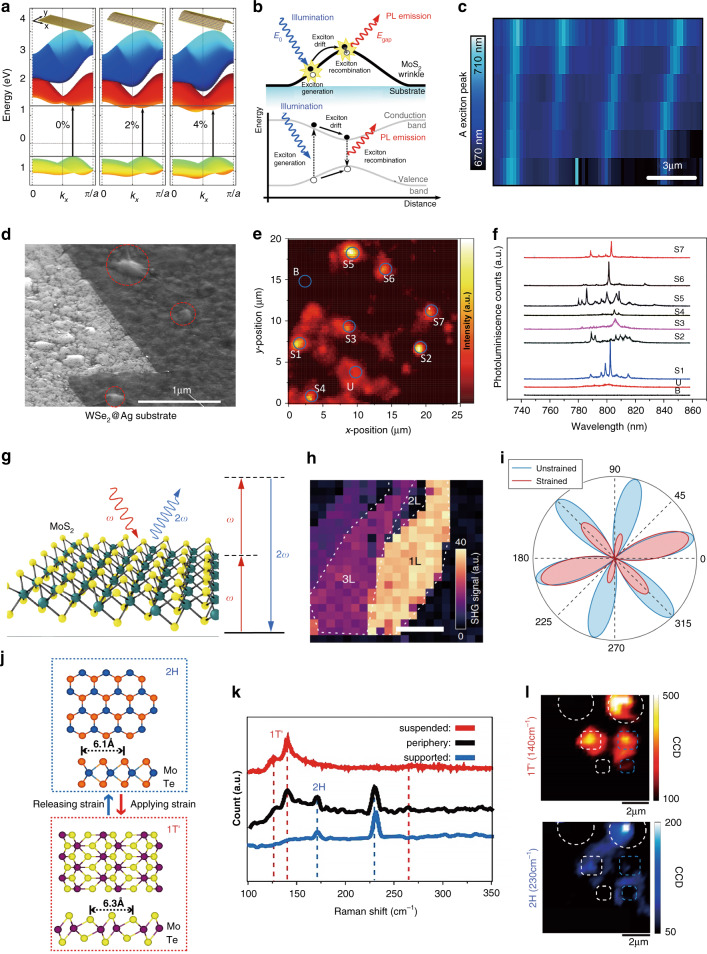
Band structure, exciton funnel effect, spontaneous emission enhancement, SHG, and phase transitions in TMDCs under local strain. **a** Calculated band structures for nonuniformly strained monolayer MoS_2_ under 0% (left panel), 2% (middle panel), and 4% (right panel) strian^41^. **b** Schematic of the exciton funnel effect induced by the inhomogeneous strain in a wrinkled MoS_2_ region^41^. **c** Spatial distribution of the A exciton wavelength in a four-layer thick MoS_2_ flake with four wrinkles^41^. **d** SEM micrograph of a WSe_2_ monolayer conformally coated on a rough silver surface, where red dotted circles denote strained regions on silver nanoparticles^80^. **e** Spatially resolved and wavelength integrated (750−850 nm) PL intensity map of the sample in **c**^80^. **f** PL spectra taken at the locations circled in **e**. The spectra labeled U and B are for the pristine monolayer and bare substrate^80^. **g** Schematic of the SHG process in strained MoS_2_ (ref. ^81^). **h** SHG intensity maps for monolayer (1L), bilayer (2L), and trilayer (3L) MoS_2_ on a Si/SiO_2_ wafer^81^. **i** Polarization-resolved SHG intensity patterns for a strained (red) and unstrained (blue) MoS_2_ monolayer^81^. **j** Schematic of the strain-induced reversible phase transition in MoTe_2_ (ref. ^86^). **k** Raman spectra taken at the suspended (red), peripheral (black), and supported (blue) areas in a cavity-supported MoTe_2_ thin film with an initial phase of 2H (ref. ^86^). **l** Raman intensity maps at the 140 cm^−1^ (1T′ phase, top panel) and 230 cm^−1^ (2H phase, bottom panel) peaks^86^. Reprinted **a**–**c** with permission from ref. ^41^. Copyright (2013) American Chemical Society. Reprinted **d**–**f** with permission from ref. ^80^. Copyright (2018) American Chemical Society. Reprinted **g**–**i** with permission from ref. ^81^. Copyright (2018) Springer Nature. Reprinted **j**–**l** with permission from ref. ^86^. Copyright (2016) American Chemical Society

Apart from homogenous in-plane strains, local inhomogeneous strains can also be induced by local strain engineering, providing other exciting avenues for tailoring distinctive optical properties of 2D materials on the nanoscale. As a concrete example, the electronic band structures of a zigzag MoS_2_ ribbon calculated using the TB model with periodic boundary conditions are plotted in Fig. 3a. The corresponding bandgap decreases as the strain increases, modifying the electronic band structure on the nanoscale. As a result, the excitons drift hundreds of nanometers to the lower bandgap regions on the top of the wrinkles before recombination, as depicted in Fig. 3b; this is referred to as the “funnel effect”. The PL spectrum of the A exciton on top of four different wrinkles (Fig. 3c) demonstrates that the A exciton can be confined by local strains^41^. The funnelling of photogenerated excitons toward regions of higher strain has potential for many applications in diverse fields, such as single-photon sources^80^ for quantum networks and communications and solar cells^44^ for photovoltaic devices.

When a homogeneous strain is applied to two- or three-layer TMDCs, a rapid increase in the PL intensity can be observed (Fig. 2f, e), which can be attributed to the transition from an indirect to direct band structure. In addition, TMDCs can be designed as ultracompact quantum light emitters under local strain (due to tight exciton localization) to produce large spontaneous emission enhancements and very sharp spectral lines at several wavelengths. For example, depositing monolayer WSe_2_ on a rough metal (Ag) surface creates strain-induced quantum emitters at Ag islands and nanoparticles (as shown in Fig. 3d), and their emission is largely enhanced by the localized surface plasmon resonances (LSPRs) of the Ag nanostructures through the Purcell effect^80^. A finite-difference time-domain simulation showed an ~30-fold field enhancement at the tip of a Ag nanocone excited by a dipole source, which induces large decay rate enhancements. The emission lines (Fig. 3f) corresponding to selected positions on the WSe_2_ on the rough Ag surface (Fig. 3e) show very sharp PL peaks (linewidths much narrower than those of the unstrained material). This phenomenon motivated the rapid growth of research on TMDC-based single-photon emission on metallic substrates.

### Strain-induced second-harmonic generation in TMDCs

Second-harmonic generation (SHG) is a second-order nonlinear optical process, in which two photons with the same frequency are combined into a single photon in a noncentrosymmetric medium (within the electrostatic approximation); see monolayer MoS_2_ in Fig. 3g. An example of SHG in TMDCs is illustrated in Fig. 3h (ref. ^81^), where a MoS_2_ monolayer exhibits giant second-order nonlinear polarizability due to the breaking of spatial inversion symmetry; this makes the MoS_2_ monolayer a good platform for studying SHG. Inspired by the close relationship between the SHG signal and the crystal lattice, SHG mapping can be exploited to quickly and nondestructively identify the strains in 2D TMDCs^82^.

We now discuss how the SHG signal can be used to sense strain in TMDC monolayers. The second-order nonlinear susceptibility tensor $\upchi_{klm}^{(2)}$ of 2D materials, dependent on strain, can be written (to lowest order) as^81,83^

8 $${\upchi}_{klm}^{\left( 2 \right)} = {\upchi}_{klm}^{\left( {2,0} \right)} + {\mathbf{p}}_{klmij}{\upvarepsilon }_{ij}$$

where ${\mathbf{p}}_{klmij} = \frac{{\partial \upchi_{klm}^{\left( {2,0} \right)}}}{{\partial {\boldsymbol{\varepsilon }}_{ij}}}$ and $\upchi_{klm}^{\left( {2,0} \right)}$ describes the second-order nonlinear susceptibility of the unstrained crystal. **p**_*klmij*_ is the fifth-rank photoelastic tensor, which relates strain *ε*_*ij*_ to the nonlinear susceptibility. Given the symmetric strain tensor (*ε*_*ij*_ = *ε*_*ji*_) and dispersion-free SHG process, the photoelastic tensor of monolayer TMDCs should possess the following symmetries: **p**_*klmij*_ = **p**_*kmlij*_ = **p**_*klmji*_ = **p**_*kmlji*_. Since hexagonal TMDC monolayers have *D*_3h_ lattice symmetry, considering these symmetries, the 2D TMDC monolayer photoelastic tensor should have 12 nonzero elements that are functions of (only) two parameters, *P*_1_ and *P*_2_ (ref. ^81^). The induced second-order nonlinear polarization can be described by ${\mathbf{P}}_k^{\left( 2 \right)}\left( {2\omega } \right) \propto$ ${{\upchi }}_{klm}^{\left( 2 \right)}E_l(\omega )E_m(\omega )$, where *E* refers to the incident electric field. For linearly polarized light, incident at angle *ϕ*, analysis of the SHG signal with the same polarization yields the SHG intensity of the form^81^:

9 $$I_\parallel ^{\left( 2 \right)} \propto \frac{1}{4}\left( {A\cos \left( {3\phi } \right) + B\cos \left( {2\theta + \phi } \right)} \right)^2$$

where ${\mathrm{A}} = \left( {1 - {\upnu}} \right)\left( {P_1 + P_2} \right)\left( {\varepsilon _{xx} + \varepsilon _{yy}} \right) + 2\chi ^0$ and ${\mathrm{B}} = (1 + {\upnu})\left( {P_1 - P_2} \right)\left( {\varepsilon _{xx} - \varepsilon _{yy}} \right)$. Note that *χ*^0^ is the nonlinear susceptibility parameter of unstrained monolayer TMDC crystals, *v* is the Poisson’s ratio, *ε*_*xx*_ and *ε*_*yy*_ denote the principal strains, and *θ* is the direction of the principal strain. Consequently, once the parameters *P*_1_ and *P*_2_ are deduced from the polarization-resolved SHG intensity at different strain levels, these two parameters can be utilized to spatially map the strain field^81^. The proof-of-concept polarization-resolved SHG intensity study demonstrated in Fig. 3i opened up a window for imaging the strain distribution below the optical diffraction limit.

### Phase transition of TMDCs engineered by strain

Many TMDCs exhibit different crystal structures (polymorphs) for various deposition or postprocessing conditions. These polymorphs retain the general MX_2_ (X-M-X trilayer) structure, but exhibit different chalcogen coordination structures around the transition metal atoms. The most common structures are the 2H (trigonal prismatic coordination) phase, 1T (octahedral coordination) phase, and 1T′ (distorted octahedral coordination) phase^84^. For group-VI TMDC monolayers, theoretical predictions showed that the most stable phase under ambient conditions is 2H (except for in WTe_2_^85^)—these materials are semiconductors with bandgaps in the 1–2 eV range. On the other hand, 1T and 1T′ tend to be metallic. The coexistence of metallic and semiconducting TMDC monolayer polymorphs has spurred studies of phase transitions in these systems. The existence of metal–insulator transitions under or close to ambient conditions suggests possible nonvolatile information storage applications^85^.

A strain-driven semiconductor-to-metal (2H-1T′) transition of MoTe_2_ under room temperature was reported in 2016 (ref. ^86^; such a reversible phase transition is shown in Fig. 3j). Given that the 2H and 1T′ phases of monolayer MoTe_2_ present different Raman signatures and peaks (140 cm^−1^ for the 1T′ phase and 230 cm^−1^ for the 2H phase), the Raman spectra could be used to identify the occurrence of phase transitions (Fig. 3k). In this experiment, a thin film of MoTe_2_ was transferred onto a substrate patterned with cavities of different diameters. The MoTe_2_ monolayers suspended over the cavities were then subjected to external tensile strains using AFM tips. Evidence of a phase transition from 2H to 1T′ was only observed in the suspended regions. These suspended areas showed 1T′ Raman signals (140 cm^−1^ in the top panel of Fig. 3l), while only 2H MoTe_2_ signals were found in the supported (unstrained) areas (230 cm^−1^ in the bottom panel of Fig. 3l). The application of strain reduced the phase transition temperature from 855 °C to room temperature in this experiment. Thus, phase-change-related TMDC electrical and optical property manipulation at room temperature may find applications in extremely sensitive optical and electrical sensors^86^.

## Optical behavior of unstrained graphene

**Fig. 4:**
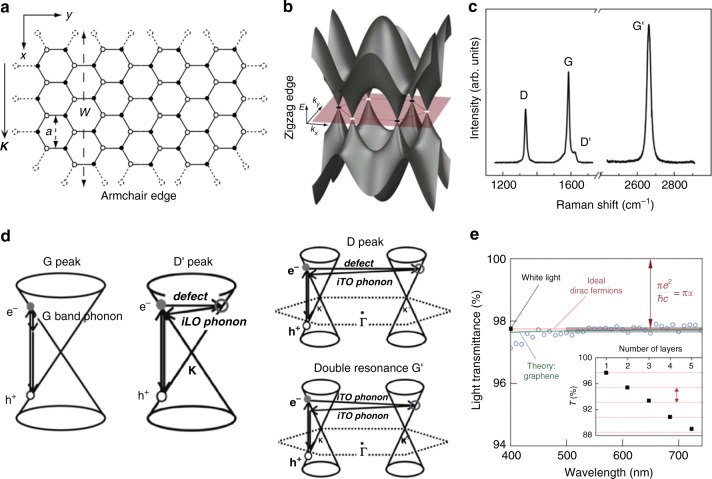
Crystal and band structures, Raman responses, and optical transmission of unstrained graphene. **a** Honeycomb lattice of graphene, and **b** its band structure^90^. **c** Raman spectrum taken at a graphene edge^7^. **d** Schematic of the formation of different Raman resonances in graphene^7^. **e** Transmittance spectrum of single-layer graphene (open circles), with the gray bar representing the standard error of measurements. The red line is the transmittance spectrum given by *T* = (1 + 0.5*πα*)^–2^ expected for 2D Dirac fermions, whereas the green curve is given by the theory taking into account the nonlinearity and warping effects in the electronic spectrum of graphene. The inset shows the transmittance of white light as a function of the graphene layer number (squares). The dashed lines represent an intensity reduction of *πα* for each added layer^89^. Reprinted **a** and **b** with permission from ref. ^90^. Copyright (2008) by the American Physical Society. Adapted **c** and **d** from ref. ^7^. Copyright (2009) with permission from Elsevier, from ref. ^89^. Reprinted **e** with permission from AAAS

Many intriguing physical properties of graphene can be understood by applying fundamental knowledge of topology^87^, differential geometry^88^, and quantum electrodynamics^89^. Graphene is composed of carbon atoms arranged in a hexagonal structure, as shown in Fig. 4a. This structure can be described as a triangular lattice with a two-atom basis (labeled black points and white points), which can be called A atoms and B atoms. In this structure, there is contact between the bonding (*π*) and antibonding (π*) orbitals at each K and K′ energy valley (only considering hopping between the nearest-neighbor atomic sites). The A (B) atoms generate a triangular A (B) sublattice that can be considered as an additional degree of freedom, namely, “pseudospin”^90^. Calculations on pristine graphene showed that the electronic band structure exhibits a linearly tapered dispersion relationship in the vicinity of the K and K′ points (Fig. 4b)^90^. The zero bandgap at these Dirac (K, K′) points endows graphene with peculiar electrical properties, e.g., an outstanding carrier mobility. Compared to the high absorptivity of TMDC monolayers (~15%)^91^, intrinsic graphene exhibits zero bandgap at the Dirac points, and a poor out-of-plane absorptivity of ~2.3% (ref. ^89^). These properties can be modified by doping to tune the Fermi level^92^, or by stacking graphene and dielectric layers (via self-assembly) to form a metamaterial grating structure that transforms incident spatial light into waveguided modes^93^ (where the in-plane absorption of graphene can be up to 10 dB due to the large area covered by graphene with a loss of 0.23 dB/µm (ref. ^94^)). In this section, we review some peculiar optical properties of unstrained graphene.

### Raman spectroscopy of unstrained graphene

Because of its distinctive electronic properties, Raman spectroscopy is the most effective technique for illustrating the optical properties of graphene. We begin this section by considering the phonon dispersion of graphene, which is essential to understand the Raman spectra. The six phonon modes existing in graphene can be classified into three types: two in-plane optical phonons, two in-plane acoustic phonons, and two out-of-plane phonons (one optical and the other acoustic). Several peaks in the Raman spectrum of graphene originate from these different phonon modes, such as G, D, and G′ (also called 2D in some articles).

The G peak is caused by the in-plane vibration of *sp*^2^-bonded carbon; it is associated with the doubly degenerate phonon mode, in which the in-plane optical phonons vibrate parallel and perpendicular to the A–B carbon atom bond (Fig. 4a) at the center of the BZ. The D peak is usually considered to be a disordered vibration, which depends on the incident wavelength^7^ and requires a defect to activate it^95^. Thus, the D peak can be used to characterize structural defects (including edges) in graphene. The G′ peak is the second-order Raman peak of the double phonon resonance, in which the two in-plane optical phonons vibrate perpendicular to the A–B carbon atom bond and reflects the number of layers in multilayer graphene^96^, the degree of susceptibility (elastic constant) to strain^30,97^, and the stacking order of graphene layers^7^. In addition, the weak D′ peak is related to disorder-induced intravalley inelastic scattering processes. For example, Fig. 4c shows a typical Raman spectrum of graphene measured with a laser excitation energy of 2.41 eV (ref. ^7^); the G and G′ peaks are near 1582 and 2700 cm^−1^, respectively. If multiple edges or defects exist in graphene, then the D and D′ peaks would be at ~1350 and 1620 cm^−1^, respectively. Figure 4d shows a schematic illustration of the underlying Raman peak formation physics.

### Optical transmission and absorption of unstrained graphene

The light transmission and absorption of graphene related to electron and photon interactions have attracted considerable attention. Because the high-frequency optical conductivity under the Dirac fermion approximation in graphene is universal (equal to *e*^2^/4ℏ for all $\hbar \omega \,>\, 2E_{\mathrm{F}}$, where *E*_F_ is the Fermi energy)^53,98–100^, the optical response of graphene is limited by the fine-structure constant *α* = *e*^2^/ℏ*c* ≈ 1/137, which describes the coupling between light and relativistic electrons in quantum electrodynamics^89^. For example, the optical transmittance and reflectance of graphene are given by *T* = (1 + ½*πα*)^–2^ and *R* = ¼*π*^2^*α*^2^*T*, respectively, under normal incidence. As a result, the graphene opacity ((1 − *T*) ≈ *πα*) is light frequency-independent. The experimental light transmittance spectrum in the visible range is shown in Fig. 4e. Although atomically thick graphene exhibits an absorptivity of only ~2.3% for white light^89^, its transmittance should be tunable through external strains.

## Optical effects in strained graphene

### Electronic band structure of strained graphene

Although pristine graphene exhibits distinctive electronic and optical properties, its gapless electronic band structure greatly limits its practical optoelectronic applications. However, strain engineering can be applied to produce a range of appealing properties, such as strain-induced opening of the bandgap and formation of a giant pseudomagnetic field. For uniaxial strain exceeding 20%, the bandgap of graphene opens (theoretically predicted by TB model calculations^57^). A combination of shear and uniaxial strain can open the gap from 0 to 0.9 eV under a reversible and more accessible deformation range of 12–17% (ref. ^58^). The gap opens at various points in the BZ of graphene under combined shear and uniaxial strain of *ε* = 15% except for strain application along the armchair direction^101^.

Strain also modifies the magnetic behavior of graphene. Designed distortion of the graphene lattice induces a very large, (nearly) uniform pseudomagnetic field and pseudo quantum Hall effect^46^. For example, the application of a modest strain field with triangular symmetry creates a uniform pseudomagnetic field, which can be as large as 10–10^2^ *T* in graphene^102^. Similar strain-induced pseudomagnetic fields are also predicted in TMDCs; e.g., in MoS_2_, the gaps between the Landau levels should scale as $\hbar \omega c/K_B \simeq 2.7B_0\left[ {\mathrm{T}} \right]K$ (ref. ^103^).

### Strain tuning and splitting of Raman resonances

Strain engineering in graphene has a longer history than that in TMDCs, which has enabled various applications. Depending on the straining techniques, graphene optical signals (e.g., the Raman resonance) can be indirectly modified by electrical signals^104^, gas pressure^105^, and even sound waves^106^. As a result of the modified phonon dispersion and electronic band structure, both theoretical analysis and experiments have demonstrated two common characteristics in the Raman spectra: strain-induced shifting and splitting of Raman spectral peaks (both of which depend on the direction and magnitude of the applied strain). Experimental results showed that the G and G′ bands in graphene undergo linear redshifts (blueshifts), as a function of applied tensile (compressive) strain. More specifically, when the C–C bond length or angle in graphene is changed under strain, the hexagonal symmetry of graphene is destroyed, removing the degeneracy of the two optical phonons at the Γ point in the BZ, and the G band splits into two subbands (called G^+^ and G^−^). Therefore, for sufficiently large, asymmetric, or nonuniform local strain, G band splitting can be observed^107–109^. Similar splitting of the G′ (2D) band has also been reported^30,31,97,110^.

**Fig. 5:**
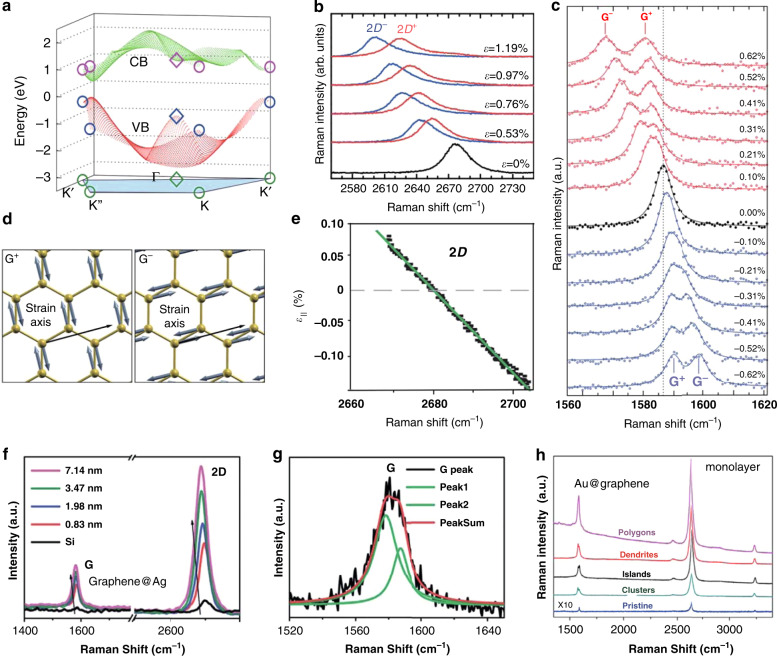
Gap opening and Raman responses of strained graphene. **a** Calculated band structure (valence band marked as VB and conduction band marked as CB) of graphene under combined shear and uniaxial strain with *ε* = 15% (ref. ^58^). **b**, **c** Raman spectra of graphene as a function of uniaxial strain, showing the evolution of the 2D (**b**)^30^ and G (**c**) bands^111^. **d** Eigenvectors of G^+^ and G^−^ modes for strained graphene calculated by density-functional perturbation theory, where G^−^ is polarized along the strain axis^109^. **e** Linear relationship between the 2D peak position and the biaxial strain in graphene^97^. **f** Raman spectra of graphene on Ag thin films, showing redshifts of both the G and 2D peaks increasing surface roughness^113^. **g** Splitting of the G peak of graphene on a rough Ag film (3.47 nm surface roughness)^113^. **h** Raman spectra of pristine graphene and graphene modified by four Au nanostructures^108^. Reprinted **a** with permission from ref. ^58^. Copyright (2010) by the American Physical Society. Reprinted **b** with permission from ref. ^30^. Copyright (2011) by the American Physical Society. Adapted **c** with permission from ref. ^111^. Copyright (2010) from American Chemical Society. Reprinted **d** with permission from ref. ^109^. Copyright (2009) by the American Physical Society. Adapted **e** with permission from ref. ^97^. Copyright (2010) from American Chemical Society. Reproduced **f**–**g** from ref. ^113^ with permission from The Royal Society of Chemistry. Reprinted **h** from ref. ^108^. Copyright (2013) with permission from Elsevier

We now present some representative experimental results on Raman spectral peak shifting and band splitting. The results of a typical experiment are illustrated in Fig. 5b, c, which show both shifting and splitting of the G and G′ (2D) bands. Figure 5d shows a schematic of the orthogonal eigenvectors of the G^+^ and G^−^ modes, as determined from density-functional perturbation theory^109^. The redshift coefficients for the G′ (2D), G^+^, and G^−^ bands under tensile strain are −64 cm^−1^/%, −10.8 cm^−1^/%, and −31.7 cm^−1^/%, respectively^109^. Interestingly, splitting of the G band and a blueshift also occur when graphene is subjected to compressive strain (Fig. 5c)^111^. The magnitude of the shift and G band splitting is more severe under tension than compression (Fig. 5c), as indicated by the sub-band shift coefficients (−31.4 cm^−1^/% for the G^+^ band and −9.6 cm^−1^/% for the G^−^ band under tension; 22.3 cm^−1^/% for the G^+^ band and 5.5 cm^−1^/% for the G^−^ band under compression). In some specific experiments (without band splitting), the shift coefficient for the G′ band is much larger than that for the G band (−27.8 cm^−1^/% for the G′ band and −14.2 cm^−1^/% for the G band under uniaxial tension^112^; −160.3 cm^−1^/% for the G′ band and −57.3 cm^−1^/% for the G band under biaxial tension^97^). With increased strain magnitude, the 2D (G′) peak also splits into two peaks (labeled 2D^+^ and 2D^−^) under homogeneous uniaxial strains (Fig. 5b); this is associated with anisotropic modifications of the phonon dispersion and distorted Dirac cones.

A significant enhancement of Raman signals, i.e., surface-enhanced Raman scattering (SERS), can be observed when graphene is placed on nanoscale rough metal surfaces, or vice versa. Two main physical mechanisms are responsible for this phenomenon. On the one hand, the LSPRs of subwavelength metal nanoparticles can confine and enhance the incident electromagnetic fields at the dielectric/metal interface. On the other hand, nanoparticles can induce local inhomogeneous strains in graphene that further modify the phonon dispersion. Here, two examples are introduced to elucidate these phenomena. First, graphene is placed on corrugated silver films with different surface roughnesses (Fig. 5f, g; ref. ^113^). From these figures, it can be concluded that the peaks of the G and 2D bands are enhanced and redshifted (Fig. 5f), and that the G band splits (Fig. 5g) with increasing roughness amplitude (in the 0.83–7.14 nm range). The second example^108^ concerns the Raman spectra of pristine graphene, and four different morphologies of Au nanostructures deposited on graphene (Fig. 5h), where morphology-dependent Raman intensity enhancement and G band splitting are also observed. These two examples indicate that the Raman response of graphene can be controlled by both local electromagnetic environment and strain engineering.

### Anisotropic optical response of strained graphene

**Fig. 6:**
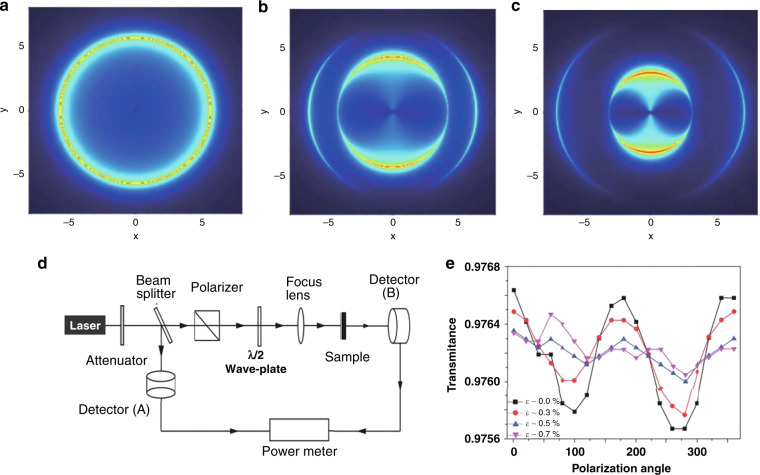
Strain-induced anisotropic optical effects in graphene. **a**–**c** Polar plots of the longitudinal optical conductivity as a function of frequency (*ω* > 0, polar axis) and electric field orientation *ϕ* (polar angle). Strain is applied along the armchair direction, and increases from **a** to **c** (*ε* = 0, 0.075, and 0.175)^47^. **d** Schematic of the absorption measurement setup for graphene^114^. **e** Polarization-resolved optical transmittance of strained graphene^114^. Reprinted **a**–**c** with permission from ref. ^47^. Copyright (2010) by American Physical Society. Reprinted **d** and **e** from ref. ^114^ with permission from Wiley

As discussed above, the optical transmission and absorption of unstrained graphene only depend on the fine-structure constant irrespective of the frequency or polarization of incident light in the visible spectrum. From a theoretical point of view, the striking optical properties of graphene, such as graphene plasmons and magneto-optical effects (Faraday rotation), are related to the optical conductivity. In 2010, Pellegrino et al. studied strain-induced variation in the optical conductivity of graphene. In this work, calculations were performed within the TB approximation to predict the frequency dependence of the longitudinal optical conductivity as a function of strain^47^. They reported the longitudinal optical conductivity (determining the optical absorption) as a function of the frequency (polar axis) and polarization (polar angle) of incident linearly polarized light for different applied uniaxial strains (Fig. 6a–c)^47^. They observed that the optical conductivity is isotropic and exhibits a maximum at the Van Hove singularity under vanishing strain (Fig. 6a). However, the application of strain induces anisotropy in the optical conductivity (Fig. 6b, c), implying that the optical transmission and absorption of strained graphene depend on the polarization of incident light. Later, in 2014, an experiment^114^ verified the tuneable strain-induced anisotropic optical conductivity of graphene. This optical absorption measurement setup is depicted in Fig. 6d, where the sample is strained by bending. This work directly demonstrated controllable optical anisotropy in graphene, i.e., polarization-dependent transparency, as depicted in Fig. 6e, where the tuneable optical conductivity is evidenced by the different transmittances as a function of strain. This is a new approach for manipulating the optical properties of graphene through strain engineering, possibly paving the way for new practical applications, e.g., optical isolators.

## Strain engineering techniques

In this section, we briefly review various techniques for applying strains to 2D materials. This section is divided into three parts based on the nature of the applied strain: homogeneous uniaxial strain, homogeneous biaxial strain, and inhomogeneous local strain.

### Homogeneous uniaxial strain

#### Bending

**Fig. 7:**
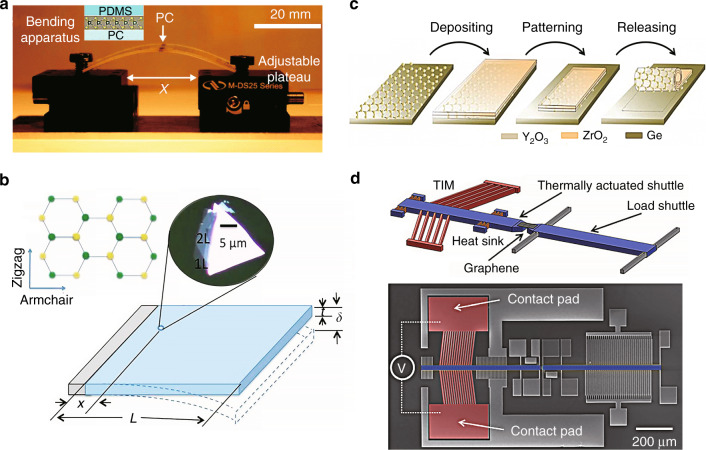
Bending and elongation for generating homogenous uniaxial strain. **a** Experimental setup for two-point bending. The insert shows a PC-MoSe_2_-PDMS stack created by placing a viscoelastic (PDMS) stamp carrying exfoliated MoSe_2_ upside down onto a polycarbonate (PC) substrate^115^. **b** Schematic diagram of cantilever bending where the (1L or 2L) MoS_2_ sample is placed near the fixed edge of the flexible PMMA substrate (see the inset)^29^. **c** Schematic of rolling two transparent oxide layers stacked on a Ge substrate^118^. **d** Schematic of a MEMS device for generating large strains in graphene (upper panel) and its SEM micrograph (lower panel)^119^. Reprinted **a** from ref. ^115^. Published by The Royal Society of Chemistry. Reprinted **b** with permission from ref. ^29^. Copyright (2013) American Chemical Society. Reprinted **c** from ref. ^118^ with permission from Wiley. Adapted **d** with permission from ref. ^119^. Copyright (2014) American Chemical Society

Controllable homogeneous uniaxial strain can be applied to a 2D material via bending. This is usually accomplished by transferring the 2D material to a flexible substrate (PMMA^29^, PC^74^, PDMS^115^, etc.) followed by bending the substrate, as depicted in Fig. 7a, b. One surface of the bent, flexible substrate will be in compression, and the other in tension, resulting in a uniform, uniaxial strain (the uniaxial strain is perpendicular to the axis of bending) on the 2D material on the substrate surface. We can divide such techniques into two main categories based on the apparatus used: two-point bending systems (Fig. 7a) and cantilever bending systems (Fig. 7b). The uniaxial strain induced on the surface of a flexible substrate in two-point bending is^115^

10 $$\varepsilon = \frac{t}{{2R}}$$

where *t* is the substrate thickness and *R* is the radius of curvature (assuming *R* ≫ *t*)^109^. For a cantilever of length *L*, the uniaxial strain on the substrate surface is^29^

11 $$\varepsilon = \frac{{3t\delta }}{{2L^2}}\left( {1 - \frac{x}{L}} \right)$$

where *x* is the distance from the fixed edge of the substrate and *δ* is the deflection of the bendable edge, assuming that *δ* is small such that the maximum slope is ≪1 (ref. ^116^).

Normally, two-point bending systems are commonly employed for large 2D sheets, while the cantilever approach is applied when the 2D sheet is small. Commonly, large means that the lateral extent is ≥100 µm (Fig. 7a), whereas small means ≤10 µm (Fig. 7b). These methods have been widely applied in studies of graphene and TMDCs^28,29,74,75,78,109–111,114,115,117^. Depending on the strength of the 2D sheet and the equipment, the maximum uniaxial strain achieved by these methods is 0.5–3.8%.

#### Rolling

Because of their high strength, 2D materials can be readily rolled up. For example, many nanofabrication processes for graphene employ rolling techniques, which result in a uniaxial strain perpendicular to the rolling axis, as shown in Fig. 7c (ref. ^118^). This approach can be used to apply tensile or compressive strains depending on the pre-straining. Typical maximum compressive strains are on the order of −0.3% (ref. ^118^; a larger magnitude than most other techniques).

#### Elongation

Conceptionally, elongation is the simplest approach for applying a uniaxial homogeneous strain to a 2D material^77,112,119,120^. The 2D material is placed on the surface of a substrate, which is elongated in a tensile test device. The tensile strain in this case is easily controllable via the application of a load to the substrate. This method can routinely achieve a much higher maximum strain level (~ 4%)^77^ than those achieved via the above techniques. A MEMS device was recently^119^ fabricated to achieve uniaxial strains in graphene in excess of 10% (Fig. 7d). In this experiment, two suspended shuttle beams are bridged by graphene, one of which is thermally actuated, while the other is affixed to springs to measure the pulling force. When an external power is applied to the contact pad, current flows through the thermal shuttle beam to induce Joule heating, which expands the beam, and thereby actuates the shuttle to uniformly apply uniaxial strain to the 2D material sample^119^.

### Homogeneous biaxial strain

#### Thermal expansion

**Fig. 8:**
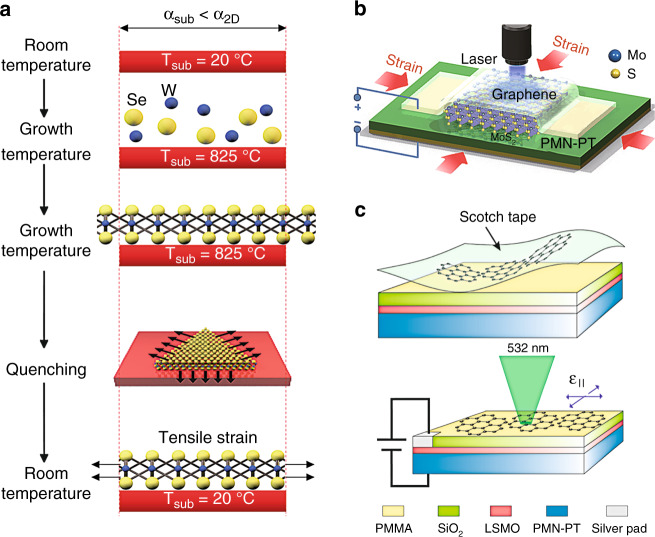
Thermal expansion and piezoelectric effect for generating homogeneous biaxial strain. **a** Schematic of the differential thermal expansion induced homogeneous biaxial tensile strain in monolayer WSe_2_, whose thermal expansion coefficient is larger than that of the substrate^121^. **b** Schematic diagram of MoS_2_ sandwiched between a piezoelectric PMN-PT substrate and a graphene layer serving as an electrode^76^. **c** Schematics of scotch-tape-exfoliated graphene on a PMN-PT substrate (upper panel) and an electromechanical device applying an in-plane biaxial strain to graphene (lower panel)^97^. Reprinted **a** with permission from ref. ^121^. Copyright (2017) Springer Nature. Reprinted **b** with permission from ref. ^76^. Copyright (2013) American Chemical Society. Reprinted **c** with permission from ref. ^97^. Copyright (2010) American Chemical Society

A biaxial strain can be applied using the idea of differential thermal expansion (Fig. 8a (ref. ^121^)). This requires a large difference in the thermal expansion coefficients of the 2D materials and the substrate. In the example, in Fig. 8a, the thermal expansion coefficient of the 2D material exceeds that of the substrate. When the 2D material is strongly adhered to the substrate and the system if heated or cooled, the expansion difference between these materials generates a homogeneous tensile biaxial strain^79,121^. While the thermal expansion coefficient is a symmetric second-rank tensor, the thermal strain will be (balanced) equal in two orthogonal directions if the substrate is amorphous, cubic, or has a (0001) surface in a hexagonal crystal or a (001) surface in a tetragonal crystal. There are two obvious deficiencies of this method: (1) the modest magnitude of the induced strain (a few tenths of a percent) and (2) the difficulty in studying the temperature dependence of a property that depends on strain.

#### Piezoelectric straining

Piezoelectric materials are strained under the application of an applied external electric field. They can serve as substrates suitable for applying both tensile and compressive strains on attached 2D materials^76,97,104^. The working principle, together with a schematic, is shown in Fig. 8b, c, where a 2D material is deposited on a hybrid substrate incorporating a PMN-PT layer, whose thickness changes upon application of an external applied electric field. Under an appropriate electrical bias, the substrate elongates in the vertical direction and compresses in the horizontal direction, which in turn applies a homogeneous in-plane biaxial compressive strain to the 2D materials (Fig. 8b)^76^. When the bias direction is reversed, tensile strain can also be applied (Fig. 8c)^97^. In this way, application of an electric field can continuously tune the magnitude of the strain (and its direction) in the −0.2% to 0.1% range^76,97^.

### Inhomogeneous local strain

In this section, we focus on techniques for applying locally inhomogeneous strains to 2D materials, i.e., there is a spatial variation in the strain tensor. It is also of technological interest to apply strains in very small areas, e.g., for miniaturization of conventional devices suitable for higher integrity and lower power consumption.

#### Laser illumination

The laser illumination method follows a similar principle to the thermal expansion method discussed in the previous section. A high intensity, focused laser is used to efficiently heat a local region of a 2D material, resulting in a nonuniform temperature distribution (e.g., a higher temperature in the center and a lower temperature at the edges), which results in an inhomogeneous local strain in the sample via differential thermal expansion^79^.

#### Wrinkling

**Fig. 9:**
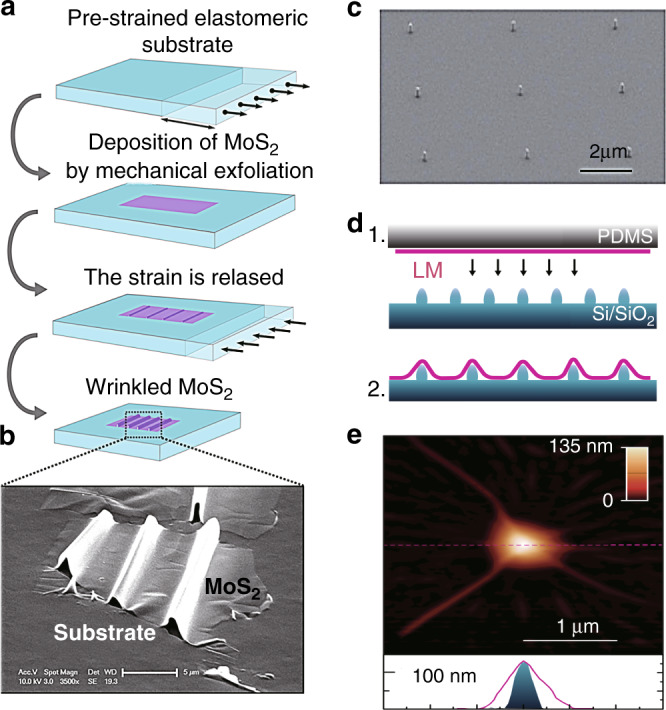
Wrinkling and nanostructure support for local strain generation. **a** Schematic of the formation of wrinkled 2D MoS_2_ (ref. ^41^), and **b** optical microscopy image of a wrinkled MoS_2_ sheet^41^. **c**, **d** SEM micrograph of an array of silica nanopillars on a silicon substrate and **d** fabrication procedures for a layered material (LM) on the nanopillars^126^. **e** AFM image of monolayer WSe_2_ on a silica nanopillar (upper panel), and height profiles of the nanopillar before (blue area) and after (pink line) LM deposition (lower panel)^126^. Reprinted **a** and **b** with permission from ref. ^41^. Copyright (2013) American Chemical Society. Reprinted **c**–**e** with permission from ref. ^126^. Copyright (2017) Springer Nature

Transferring 2D materials onto prestrained flexible elastomeric substrates (a relatively simple and broadly used method) provides a means of introducing inhomogeneous local strains into any type of 2D materials^41,122–124^. The working principle is illustrated in Fig. 9a, where an elastomeric substrate is initially stretched, 2D material sheets (e.g., exfoliated MoS_2_) are subsequently deposited on the prestrained substrate, and the load that strains the substrate is released. The large strain energy in the 2D material sample is released via localized buckling and debonding, creating a distribution of wrinkles in the 2D material (Fig. 9b). In this technique, the maximum strain accumulates on the top of the wrinkles and can be defined as^41^

12 $$\varepsilon = \pi ^2h\delta /\left( {1 - \nu ^2} \right)\lambda ^2$$

where *ε* is the uniaxial strain, *h* is the sample thickness, *δ* is the wrinkle height (distance between the top of the sample and the substrate), *λ* is the wrinkle width, and *v* is the Poisson’s ratio^125^. Of course, if the prestrain in the substrate is not uniaxial, then a more complex distribution of wrinkles and final strain states may be induced.

#### Nanostructure support

In this class of techniques, the morphology of the substrate must be patterned prior to transferring the 2D material. This can be accomplished by positioning nanopillars^126–129^, nanorods^130^, etched nanoholes^131^, or other nanostructures on a flat surface, by producing rough substrates^80,113^ or by substrate patterning via nanolithography methods before transferring the 2D materials. In all of these cases, the non-flatness of the substrate and the conformality of the 2D materials induce strains in the 2D material via bending/stretching. These types of approaches have been applied in recent years due to the rising interest in single-photon emission, which is a key element in optical quantum computation.

For the sake of clarity, two typical examples are introduced here, in which nanopillars and rough metallic surfaces are used. In the first example^126^, exfoliated monolayer WSe_2_ flakes are deposited on a substrate with a square lattice of Si nanopillars. Figure 9d shows a schematic of the formation of the inhomogeneous local strain, while Fig. 9c, e show an SEM micrograph of the Si nanopillar array and an AFM image of monolayer WSe_2_ on a Si nanopillar, respectively. In the second example^80^, a sapphire substrate coated by a thin silver film (200 ± 10 nm) followed by deposition of a 3 nm Al_2_O_3_ layer to protect the silver film from oxidation is used as a rough metallic surface to apply local strains to the later transferred WSe_2_ (ref. ^80^).

## Summary and comparison of strain-induced optical effects and strain engineering techniques

We first provide a brief summary of the optical effects induced by various types of strain (homogeneous uniaxial strain, homogeneous biaxial strain, and local inhomogeneous strain). Table 1 summarizes the spectral shift coefficients and optical properties of TMDCs and graphene under various strains. Among them, homogeneous uniaxial strain is viewed as the easiest-to-access strain technique with the highest achievable reliability, associated with the simplicity of the equipment and the ease of manipulation of the environment. Such an approach is simple to implement through bending^28,74,75,115^ or elongation^77^. Moreover, the induced uniaxial strains can be easily quantified and thus controlled. Alternatively, homogeneous biaxial strains in 2D materials can be applied in situations where harsher conditions can be sustained (e.g., high temperature^79,121^, high electrical voltage^76,104^, and high laser intensity^79^). However, such methods typically require more complex experimental instruments and more control. We note that in experiments that produce macroscopic strains, viscoelastic stamps can act as a mechanical clamp to improve the stability, and prevent interfacial sliding between the 2D material and the substrate during straining, when the interfacial adhesion is low (Figs. 7a, c and 8b). However, such an approach requires special attention to avoid the build-up of strain when the carried 2D material is transferred from the viscoelastic stamp to the stretchable substrate.

**Table 1:** Summary of different strains and corresponding optical effects.

Type of strain	Materials (max strain)	Optical effects	Refs.
Homogeneous uniaxial strain	1–2L MoS_2_ (2.2%)	−120 meV/% for 2L, −45 meV/% for 1L (PL)^a^	^28^
	1–2L MoS_2_ (0.8%)	−48 meV/% for 1L, −46 meV/% for 2L DB^b^, −86 meV/% for 2L IB^c^ (PL)	^75^
	1L WSe_2_ (1.4%)	−54 meV/% for A exciton, −50 meV/% for B exciton (Abs)^d^	^74^
	2–4L WSe_2_ (2.0%)	Indirect-to-direct bandgap transition	^78^
	1L MoSe_2_ (1.1%)	−27 ± 2 meV/% (PL)	^115^
	Graphene (0.8%)	−10.8 cm^−1^/% for G^+^ band, −31.7 cm^−1^/% for G^−^ band, −64 cm^−1^/% for 2D band	^109^
	Graphene (0.62%)	−31.4 ± 2.8 cm^−1^/% for G^+^ band, −9.6 ± 1.4 cm^−1^/% for G^−^ band	^111^
	Graphene (−0.62%)^e^	22.3 ± 1.2 cm^−1^/% for G^+^ band, 5.5 ± 1.9 cm^−1^/% for G band	^111^
	1–2L MoS_2_ (0.52%)	−64 ± 5 meV/% for 1L A exciton, −68 ± 5 meV/% for 1L B exciton, −71 ± 5 meV/% for 2L A exciton, −67 ± 5 meV/% for 2L B exciton (Abs) −48 ± 5 meV/% for 2L A exciton, −77 ± 5 meV/% for 2L IB (PL)	^29^
	1L WS_2_ (4%)	−11.3 meV/% (PL), direct-to-indirect bandgap transition at 2.5%	^77^
Homogeneous biaxial strain	3L MoS_2_ (−0.2%)	−300 meV/% (PL)	^76^
	Graphene (−0.15%)	160.3 cm^−1^/% for G′ band, −57.3 cm^−1^/% for G band	^97^
	1L WSe_2_ (−0.2% to 1.0%)	N/A	^121^
	1L MoS_2_ (0.2%)	−0.42 meV K^−1^ (PL)	^79^
Inhomogeneous local strain	1L MoS_2_ (N/A)	−0.114 meV kW^−1^ cm^−2^ (PL)	^79^
	3–5L MoS_2_ (2.5%)	Total −90 meV change for DB (PL)	^41^
	2–4L WS_2_, 2–4L WSe_2_ (1% for 3L, 2% for 4L)	Locally enhanced PL intensity with bandgap patterning	^122^
	1L WSe_2_ on Ag film (N/A)	Strong emission enhancement and single-photon emitter	^80^
	1–2L WSe_2_ on substrate (N/A)	Strong emission enhancement and single-photon emitter	^127^
	Graphene on Ag film (N/A)	Strong emission enhancement and splitting of G band	^113^
	Au deposited on graphene (N/A)	Strong emission enhancement and splitting of G band	^108^

^a^Photoluminescence.

^b^Direct bandgap.

^c^Indirect bandgap.

^d^Absorption.

^e^“−” indicates compressive strain.

Apart from the techniques for generating homogeneous strain, new approaches for inducing spatially inhomogeneous strains are gaining popularity for application on scales down to tens of nanometers, e.g., wrinkling 2D materials or depositing 2D materials on nanostructures. These approaches include the use of gels as an elastomeric substrate to realize buckling-induced delamination^41^, where a viscoelastic stamp is used as a sample carrier (to transfer the 2D material to the nanostructure) that can later be washed away (or peeled off)^80,108,113,122,127^. The local strains induced by these methods are usually much stronger, leading to a significant enhancement of the local photon emission and long lifetimes of excited electrons. Because of these characteristics, such methods are finding increasing uses in the field of 2D-material-based quantum emitters^80,127^. On the other hand, these methods suffer several disadvantages, including the costly and challenging nanofabrication process, and the lack of an effective model to quantify the local strains.

## Applications of optical properties induced by strained 2D materials

Ultrathin 2D materials, with excellent mechanical stability and electronic properties, can be exploited to produce high-sensitivity optical resonators and flexible electronic devices (and as reinforcement additives in composite materials to improve the mechanical strength). In addition, the exotic chemical, optical, and electrical properties of strained 2D material devices that exploit these properties (such as optical and mechanical sensors, and piezoelectric nanogenerators) are receiving increasing attention across a diverse set of technologies. Many of the mechanical applications of 2D materials have been reviewed recently^132^; here, we present a brief overview of recent advances in the potential and practical photonic applications of 2D materials (particularly TMDCs and graphene).

### Graphene- and TMDC-based strain sensors

**Fig. 10:**
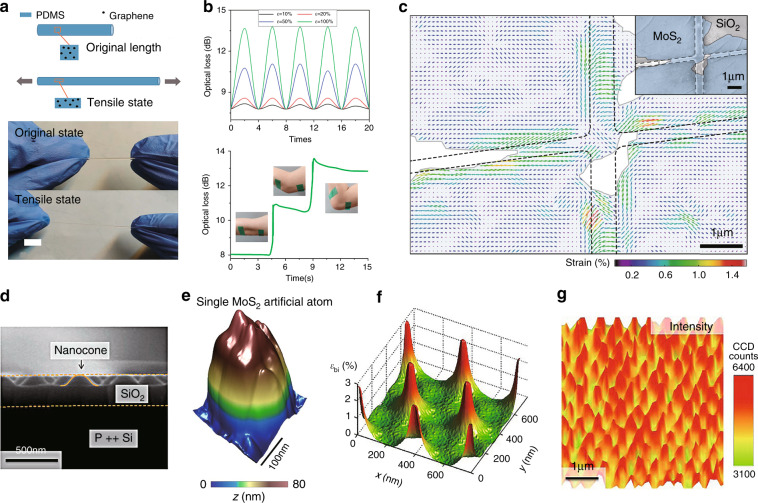
Strain sensors, SHG-based strain mapping, and broad-spectrum solar energy funnel. **a** Schematic of a PDMS fiber incorporating graphene nanocomposites (top panel) and photographs of the hybrid fiber in the original state and tensile state (bottom panel)^133^. **b** Strain-dependent optical loss of the fiber in **a** (top panel) and that measured with the movement of the human body (bottom panel)^133^. **c** Strain mapping in MoS_2_ by SHG spectroscopy. The inset shows an SEM micrograph of the sample^81^. (Scale bars in both images are 1 μm.) **d** Cross-sectional SEM micrograph of silica nanocones on a SiO_2_/Si substrate (Scale bar is 500 nm)^134^. **e** STM topography of a single MoS_2_ “artificial atom” consisting of monolayer MoS_2_ supported by a single silica nanocone (Scale bar is 100 nm)^134^. **f** Calculated local strain distribution in strain-textured MoS_2_ on the nanocone substrate, and **g** its PL peak intensity map^134^. The scale bar in **g** is 1 μm. Adapted **a** and **b** from ref. ^133^. Published by MDPI AG. Reprinted **c** with permission from ref. ^81^. Copyright (2018) Springer Nature. Adapted **d**–**g** with permission from ref. ^134^. Copyright (2015) Springer Nature

Wang et al.^133^ recently developed a strain sensor based on an optical fiber that incorporates graphene powder in PDMS. In this device, the high elastic limit of PDMS endows this fiber with excellent mechanical properties and strain capacity up to 100% strain. The working principle of this hybrid fiber in the unstrained and strained states is schematically depicted in the upper panel of Fig. 10a, and a practical device is shown in the lower panel. Researchers have shown that the optical loss of this fiber sensor is proportional to strain and that this relationship is maintained over many cycles, demonstrating the robustness and reliability of this device (optical measurements are shown in the top panel of Fig. 10b). These measurements show a maximum loss (under 100% strain) of four times that at small (~10%) strain. This range enables application of this sensor for measuring the movement of the human body; the variation in the optical loss induced by elbow bending is shown in the lower panel of Fig. 10b. The ability to sense human body motion will enable applications in human body health monitoring systems, human–machine interactions, and wearable devices. In addition, in 2018, Mennel et al.^81^ reported a powerful strain sensing technique based on the strain-induced SHG from MoS_2_ monolayers, which has the potential to enable the extraction of the full strain tensor with submicrometer resolution. They found that the two independent parameters (Eqs. (8) and (9)) are linearly proportional to the applied strain in two-point bending experiments (which makes data fitting easy). After determination of the two parameters, researchers can use SHG spectroscopy to probe spatially varying (inhomogeneous) strains in MoS_2_. A typical result obtained by this high-spatial-resolution (280 nm) strain imaging technique is shown in Fig. 10c. This approach supplements and extends optical strain detection techniques, and is expected to find use in imaging transient crystal deformations on a sub-picosecond timescale.

### Artificial atom-based broad-spectrum solar energy funnel

In 2012, Feng, et al.^44^ showed (theoretically and numerically) that inducing local elastic strains provides an accessible method to continuously decrease the bandgaps of MoS_2_ monolayers. This suggests the idea of “artificial atoms” composed of a pressure-sensitive MoS_2_ monolayer to absorb a wide range of the solar spectrum from 2 to 1.1 eV when the applied biaxial strain increases from 0 to 9%. Photovoltaic devices with such strained MoS_2_ monolayers with a continuously varying bandgap profile can capture photons over a wide range of the solar spectrum. In 2015, a proof-of-concept implementation of such a photovoltaic “artificial atoms” device demonstrated the tunability of the optical bandgap of MoS_2_ via local strains. An SEM image of this device and an STM image of an “artificial atom” are shown in Fig. 10d, e, respectively^134^. Figure 10f shows the corresponding simulation of the local strain distribution, and Fig. 10g exhibits the scanning PL map with peak intensities of the strained MoS_2_. The subsequent measurements in this work demonstrated achievement of broadband optical absorption from 677 nm (pristine MoS_2_) to 905 nm (the most highly strained MoS_2_), covering the whole visible spectrum and the most intensive wavelengths of the solar spectrum.

### Single-photon emitters

Single-photon emitters with long excited state lifetimes and spin coherence times (nanosecond scale) enable promising applications in quantum computation^135^. In semiconductor single-photon emitters, the allowed quantum interface between stationary spin qubits and propagating single photons makes 2D materials promising candidates for quantum information processing. Many experiments have demonstrated the formation of 2D TMDC-based single-photon sources^136–138^. In these experiments, the emission linewidth of the quantum dot-like defects in TMDC monolayers is much narrower (typically 0.1 meV) than that of free excitons (10 meV). Weak electrical^136^ and finite magnetic field^137,138^-controlled single-photon emissions have also been demonstrated for these artificially engineered TMDC defects. 2D material-based single quantum emitters possess more degrees of freedom for external tuning than their bulk material counterparts.

**Fig. 11:**
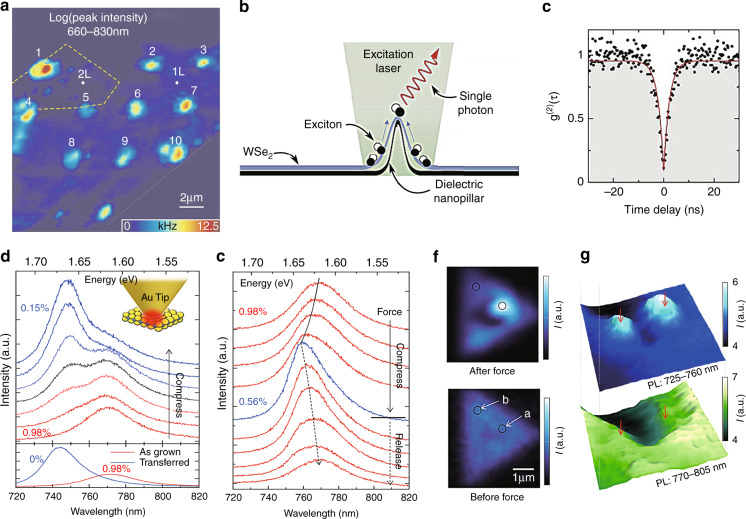
Strained TMDC-based single-photon emitters and high-resolution nanoimaging. **a** PL map of locally strained monolayer WSe_2_^127^. **b** Schematic of the scheme to realize a strain-induced quantum emitter through local deformation atomically thin WSe_2_ by a nanopillar to achieve a point-like elastic strain perturbation^127^. **c** Second-order photon correlation statistics from a single quantum emitter in **a**, revealing evident antibunching [*g*^(2)^(0) = 0.07 ± 0.04 and *τ* = 2.8 ± 0.2 ns]^127^. **d** Irreversible evolution of the TEPL spectra of an as-grown WSe_2_ monolayer with built-in tensile strain (upper panel) and PL spectra for the as-grown (red), and transferred strain-relaxed (blue) WSe_2_ monolayers (lower panel)^140^. **e** Reversible evolution of the TEPL spectra of the as-grown monolayer WSe_2_ (ref. ^140^), and **f** confocal PL images of the sample before and after applying a force. “a” denotes an irreversible area and “b” denotes a reversible area^140^. **g** Blueshifted TEPL (725–760 nm) and main TEPL (770−805 nm) images of two strain-released areas in a WSe_2_ flake near nucleation sites^140^. Reprinted **a**–**c** with permission from ref. ^127^. Copyright (2017) Springer Nature. Adapted **d**–**g** with permission from ref. ^140^. Copyright (2016) American Chemical Society

Strained monolayer and bilayer WSe_2_-based quantum light sources on an array of dielectric nanopillars were recently reported (Fig. 11a–c)^127^. The enhancement of the spontaneous emission from the strained area (compared to the pristine area) is seen in Fig. 11a, and the second-order photon correlation shown in Fig. 11c unambiguously demonstrates the single-photon emitting behavior of the locally strained WSe_2_. The authors attributed this single-photon quantum emission to the funnel-like effect in the strained region (Fig. 11b). More interestingly, the PL measurements show a stable linewidth with a much longer decay time (compared to the pristine region) of 2.8 ns in monolayer WSe_2_ and 4.8 ns in bilayer WSe_2_. This work demonstrates a new paradigm for the design of single-photon emitters that exploits strain engineering; this is expected to motivate further research on TMDC-based single-photon source integration. More recently, metal surfaces have also been employed to couple surface plasmon polaritons with 2D TMDCs to enhance the single-photon emission^80,139^. The emission enhancement is mainly attributed to the very large Purcell factor (e.g., up to 551 in the Au mirror case)^139^, and results in a much shorter decay time (down to ~98 ps) compared with results for a Si substrate^127^. Finally, 2D material-based single-photon emitters are expected to show more highly tuneable optical properties (via external applied electrical field, magnetic field, local strains, and substrate effects) than their conventional counterparts (e.g., nitrogen-vacancy centers in diamond).

### High-resolution nanoimaging

A novel hybrid nano-optomechanical tip-enhanced spectroscopy and nanoimaging approach combining the tip-enhanced Raman scattering and tip-enhanced photoluminescence (TEPL) techniques was recently reported^140^. A WSe_2_ monolayer grown by physical vapor deposition possesses a large built-in tensile strain, and its PL curve is shown in the lower panel of Fig. 11d (red line) in comparison with that of a strain-relaxed monolayer (blue line). A Au tip was used to apply different forces to release the initial strain of the sample. When the force is sufficiently large, the PL suggests that the strain relaxation is irreversible (see the PL data in the upper panel of Fig. 11d, which correspond to the PL map in the bright region in the upper panel of Fig. 11f). In contrast, a small force applied by the Au tip enables reversible strain release (see the PL curves in Fig. 11e, which correspond to the dark region in the upper panel of Fig. 11f). By combining the PL curves in Fig. 11d, e, this work showed that strain can be manipulated reversibly (elastic) or irreversibly (plastic) on the nanoscale, and that TEPL (Fig. 11g) can be used to systematically investigate strained 2D material morphologies (along with their corresponding electronic and optical properties) at very high resolution (<15 nm)^140^.

### Other potential applications

2D materials are also widely used in photodetectors, excitonic LEDs, photovoltaic devices, composite materials, sensors, microelectromechanical systems, and even in applications requiring biocompatibility^132^. For example, in 2007, Bunch et al.^141^ developed an optical resonator based on a suspended graphene sheet that can be driven either electrically or optically. This structure has potential for a wide range of applications, including pressure sensors, mass sensors, and other optoelectronic devices. In addition, strained graphene also exhibits giant Faraday rotations as a result of the pseudomagnetic field, which opens up opportunities for the design of optical isolators with two strained graphene sheets^142^.

One of the main challenges in using pristine 2D materials in photonic and optoelectronic applications is determining and understanding the excitonic dissociation and related optical responses. However, there is no doubt that defects and/or strain-induced structural changes will play an important role in the performance of related devices. Thus, a deep understanding of this issue is of great importance for enabling the design of high-efficiency optoelectronic devices. To this end, the strain information easily reflected in the optical properties of 2D materials provides a good source for understanding the excitonic fine structures of 2D materials, and their optical properties in different configurations.

## Summary and outlook

This review article systematically summarizes many of the recent advances in the application of strain in 2D materials (especially TMDCs and graphene) to modify and control their unique optical properties. We first reviewed the theory of elasticity and effective low-energy Hamiltonians as powerful theoretical tools to better understand the physical properties of 2D materials under macroscopic and microscopic strains, and then examined the effect of strain on their optical properties. This review of the theoretical and experimental literature indicates that strains can be effectively manipulated to modify the electronic band structure of 2D materials to create unexpected (novel and/or exotic) optical properties (e.g., gigantic pseudomagnetic fields). We also summarized techniques for generating different types of strains along with their corresponding photonic applications.

### Challenges

Strain engineering, especially local strain engineering, remains a rapidly evolving technology and may provide some of the most promising candidates for next-generation, semiconductor optoelectronic components (in large part because of the small dimensions inherent to 2D materials). Nonetheless, further study (e.g., of exciton transport properties) is still required. To date, local strain engineering has found its greatest application in the field of quantum emitters (see the previous section), but other relevant applications are expected to be developed. Theoretical tools (DFT) widely applied to illustrate and predict 2D material properties encounter difficulties in applications, where the strain is not uniform (due to the large number of atoms involved). This indicates the need for the development of new theoretical tools that can be applied on the appropriate scale for application to strain patterning of 2D materials.

### Future perspectives

Apart from the techniques and applications presented above, other interesting work on strain engineering of 2D materials is being performed, e.g., on catalytic^143^, magnetic^144^, and electrical^145,146^ effects. Other related work on strain engineering includes localized strain associated with twin boundaries in bilayer TMDC materials^147^, and tuneable enhanced light emission and SHG from strained WS_2_-optical-fiber-nanowire structures^148^ (for environmentally robust fiber-based sensors), application of 2D van der Waals heterostructures, and superlattices of 2D materials with different (strained) optoelectronic characteristics, etc. Special attention should be paid to understanding the effects of interlayer interactions in van der Waals heterostructures^149^ when designing coherent, atomically thin superlattices for tuning the PL^150^. Undoubtedly, atomic-level 2D heterostructures are among the most promising candidates for reviving Moore’s law. The past decades have seen the application of strain as a “key” to opening the bandgap in graphene^102,112^. However, few recent experimental results suggest that this potential is practically achievable. Recently, Motla et al.^151^ has taken a step forward by applying a modulated inhomogeneous local asymmetric elastic–plastic strain to graphene using GPa-level laser shocking to induce tunable bandgaps of up to 2.1 eV (ref. ^151^). This high-energy laser shocking provides another approach for the application of large inhomogeneous strain to 2D materials. However, the photodamage caused by high-energy laser pulses requires additional attention. In conclusion, the flexibility and optical properties of 2D materials (compared to their bulky counterparts) opens the door for the development of potentially important new optical applications. 2D materials are key components in strain-engineered optoelectronic applications.
